# Electrospun Nanotubular Titania and Polymeric Interfaces for High Energy Density Li-Ion Electrodes

**DOI:** 10.1021/acs.energyfuels.3c00192

**Published:** 2023-04-11

**Authors:** Vahid Charkhesht, Begüm Yarar Kaplan, Selmiye Alkan Gürsel, Alp Yürüm

**Affiliations:** † Faculty of Natural Science and Engineering, Sabanci University, 34956 İstanbul, Turkey; ‡ Sabanci University SUNUM Nanotechnology Research Centre, 34956 Istanbul, Turkey

## Abstract

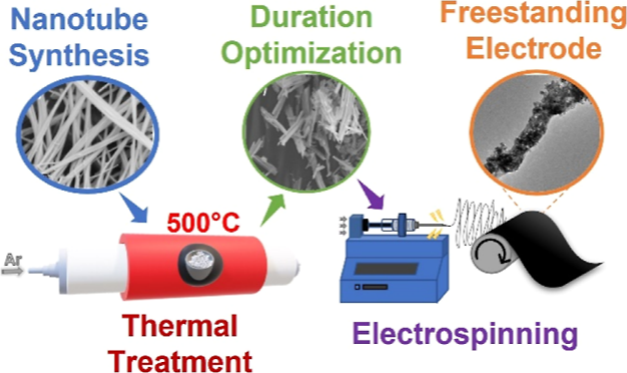

In the current study, for the first time, electrospinning of nanotubular structures was developed for Li-ion battery high energy density applications. For this purpose, titania-based nanotubular materials were synthesized and characterized. Before electrospinning with PVDF to obtain a self-standing electrode, the nanotubes were modified to obtain the best charge-transferring structure. In the current study, for the first time, the effects of various thermal treatment temperatures and durations under an Ar-controlled atmosphere were investigated for Li^+^ diffusion. Electrochemical impedance spectroscopy, cyclic voltammograms, and galvanostatic intermittent titration technique showed that the fastest charge transfer kinetics belongs to the sample treated for 10 h. After optimization of electrospinning parameters, a fully nanotube-embedded fibrous structure was achieved and confirmed by scanning electron microscopy and transmission electron microscopy. The obtained flexible electrode was pressed at ambient and 80 °C to improve the fiber volume fraction. Finally, the galvanostatic charge/discharge tests for the electrospun electrode after 100 cycles illustrated that the hot-pressed sample showed the highest capacity. The polymeric network enabled the omission of metallic current collectors, thus increasing the energy density by 14%. The results of electrospun electrodes offer a promising structure for future high-energy applications.

## Introduction

The current ecological considerations have resulted in ever-increasing demands for the substitution of fossil fuels. Seeking sustainable and low-cost energy resources has motivated researchers to enhance the efficiency of the existing alternatives. Due to the insufficient continuous power generation of solar and wind energy resources, rechargeable electrochemical energy storage systems, and particularly, Li-ion batteries (LIBs) (with an energy density of 100–265 W h/kg or 250–670 W h/L) have recently gained lots of attention. Although LIBs have been commercialized and become an intrinsic part of daily life, there is still ongoing determined research to reach safer batteries with higher energy densities in response to today’s market in portable electronics, electric vehicles, and grid storage systems.^1^

As a commercialized anode material, graphite, due to its low charging potential causes safety issues by growing dendrites. Titania-based materials thanks to their higher operational potential, not only restrain the dendrite formation but also avoid the formation of thick solid electrolyte interface (SEI) as well.^2^ Furthermore, low volume expansion during lithiation makes these materials much safer compared to other graphite-based anodes, such as silicon-added graphites.^3^

As one of the well-known derivatives of titania, hydrogen titanate (H_2_Ti_3_O_7_) nanotube (TNT) has gained attention in photocatalysts,^4^ dye adsorbents,^5^ gas sensors,^6^ and energy storage materials.^7^ Particularly, high surface area besides layered structure results in pseudocapacitive behavior providing facile fast charging capabilities in LIBs.^1^ Considering surface redox reaction as the main electron exchange mechanism during cycling, surface properties and nanotube interlayer distances play a significant role in Li^+^ diffusion. Therefore, the presence of dopants like Ag,^8^ carbon nanotubes,^9^ Sn,^10^ and N^11^ within TNT’s layered structure improves the Li^+^ mobility and results in higher capacities.

During the thermal treatment process, TNT becomes dehydrated and its orthorhombic crystal structure converts into a monolithic structure, which is called the bronze phase (TiO_2_-B).^12^ Thanks to the TiO_6_ octahedrons arrangement within the structure, TiO_2_-B possesses van der Waals gaps which enable the pseudocapacitive intercalation behavior of the cations,^13^ particularly in fast charging rates.^12^ Increasing the dehydration temperature enhances the capacity but reduces the stability of the structure. Therefore, staying at an optimized temperature for a specific duration may preserve stability and high capacity simultaneously.

The need for high-energy and -power LIBs to meet the market demands requires the reduction/elimination of the Cu or Al substrates and fabrication of the freestanding electrodes.^14,15^ Electrospinning as a scalable method is capable of providing free-standing electrodes for LIBs.^16^ In this method, by implying a high voltage current to a metallic needle of a syringe containing the electrode ink, polymeric fibers which are fully covered with active materials could be fabricated. Thereby utilizing this method, free-standing flexible electrodes can be fabricated which provides enhanced areal and gravimetric capacities.^14,17^ For instance, optimized titania-based electrospun electrodes show superior capacities because of enhanced Li^+^ diffusion through the in/inter pores.^14,15^

In this study, for the first time, the electrospinning of nanotubular structures was developed for Li-ion battery applications. Also, the study shows the impact of the duration of heat treatment at an optimized temperature.

## Experimental Section

### Materials

TiO_2_ nanoparticles (Anatase) purchased from Aldrich were used as a precursor for titanate nanotube (TNT) synthesis. To prepare the alkali solution, sodium hydroxide (NaOH) pellets supplied by Sigma-Aldrich were dissolved in de-ionized (DI) water. For controlling the pH of the solution, a hydrochloric (HCl) solution was utilized. Hydrothermal synthesis was performed using a Teflon-lined stainless-steel autoclave. Carbon black (CB) (Vulcan XC-72R) was purchased from the fuel cell store. PVDF (*M*_w_ = 380,000 g/mol) powder was purchased from Solvay. Tetrahydrofuran (THF), *N*,*N*-dimethylacetamide (DMAc, 99%), and *N*,*N*-dimethylformamide (DMF, 99%) were purchased from Sigma-Aldrich. 1 M lithium hexafluorophosphate (LiPF_6_) solution in ethylene carbonate (EC) and diethyl carbonate [EC/DEC = 50/50 (v/v)] was purchased from Sigma-Aldrich.

### Synthesis of TNT

**Figure 1:**
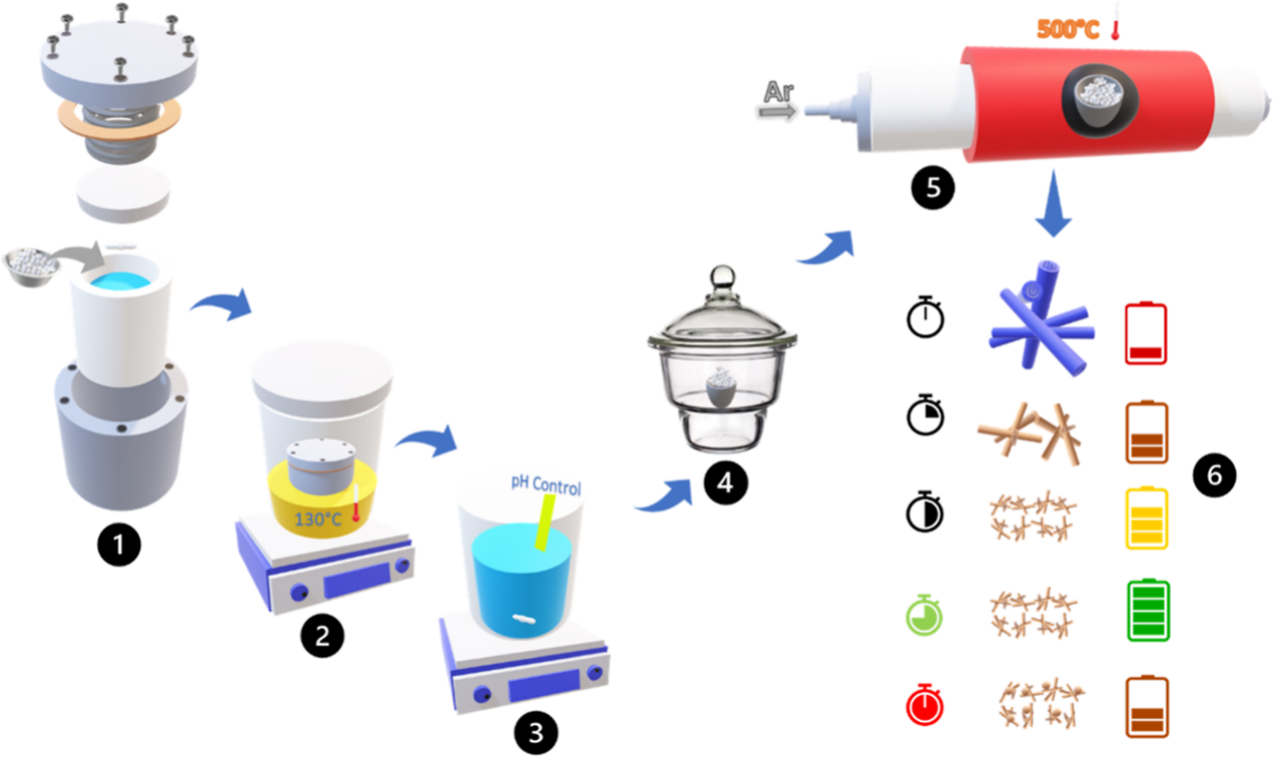
Synthesis procedure: (1) addition of anatase powder into the Teflon-lined autoclave filled with the NaOH solution. (2) Hydrothermal procedure while stirring within the oil bath. (3) Acid washing step by controlling pH. (4) Drying samples before heat samples. (5) Heat treatment of the nanotubes under an Ar atmosphere at 500 °C. (6) Investigation of the effect of various heat treatment durations on the morphology and capacity behavior of the samples.

1.5 g of TiO_2_ was mixed with a 100 mL 10 M NaOH solution and put into the autoclave. The autoclave was heated up to 130 °C and kept at that temperature for 48 h. After natural cooling, the content of the autoclave was poured into a beaker containing DI water, and 0.1 M HCl solution was added to the beaker to replace Na^+^ with H^+^. After pH control, the sample was filtered and dried at 110 °C for 24 h. The dried samples were heat treated in an Ar-controlled atmosphere at different temperatures and in various durations. Figure 1 shows the experimental procedure in detail.

### Structural Characterization

The crystal structures of the as-received, synthesized, and heat-treated powders were investigated using Bruker, D2 Phaser, X-ray diffractometer at a 2θ range of 2–90° with a 0.02 step size using Cu Kα radiation. The X-ray Rietveld refinement method was used to identify the phases contributing to the heat-treated samples. The DiffracPlus TOPAS 4.2 software package (Bruker AXS, Karlsruhe, Germany) was used to analyze the crystallographic information files of related phases existing in the Inorganic Crystal Structure Database (FIZ Karlsruhe 2019, version 4.2.0). A Zeiss LEO Supra 35VP SEM-FEG field-emission electron microscope at 3 kV operating voltage was used to investigate the surface morphology of the samples. A JEOL JEM 2000FX transmission electron microscope was utilized to analyze the morphology and interlayer distances of the nanotubes before and after the heat treatment. Nitrogen adsorption/desorption isotherm (77 K) data were used to analyze the porosity within the structure. The specific surface area was measured using adsorption/desorption values between *P*/*P*° of 0.05 and 0.35 with the Brunauer–Emmett–Teller (BET) technique. The Barrett–Joyner–Halenda method was used to measure the pore size distribution (PSD) of the active materials before and after the heat treatment by the desorption branch of the isotherm. All samples were degassed for 24 h at 60 °C before the experiment.

### Electrospinning

To obtain the binder, first, a 14 wt % PVDF stock solution was prepared by the overnight stirring of the polymer powder within a mixture of DMAc and THF solution (DMAc/THF weight ratio is 7:3). TiO_2_-B nanotubes and CB powders (with a weight ratio of 6:2) were dispersed in DMF and acetone mixture (with a weight ratio of 7:3) and sonicated for 40 min. Finally, the already prepared polymer stock solution was added to the dispersion and mixed for 24 h. The ultimate polymer/CB/TiO_2_-B weight ratio was 20/30/50. The electrospinning was performed at 70% RH, 18 kV, with a flow rate of 1 mL/h, and a needle-to-collector distance of 8 cm. The electrospun electrodes were pressed using a hydraulic pressing machine at 0.3 ton at 25 and 80 °C for 10 min to increase the fiber volume fraction within the highly porous matrix.

### Cell Assembly and Electrochemical Characterization

To prepare the cast electrodes, the electrode slurry including active material, CB, PVdF, and NMP was mixed and bladed on a copper foil followed by drying at 130 °C overnight. The cast electrodes and already prepared electrospun electrodes were cut into 15 mm diameter circles. CR 2032 coin cells were used to assemble the half cells under an argon-filled glovebox [Jacomex GP (Campus)]. The two-electrode system included Celgard PP, Li metal chips, and active material-based electrodes as the separator, the counter electrode, and the working electrode, respectively. The standard electrolyte including 1 M LiPF_6_ in EC/DEC (1:1, v/v) was used within the half cells. To record the cyclic voltammograms (CV) of the samples, a Princeton Applied Research PARSTAT MC system was used. CV tests were carried out within 1.0–3.0 V (vs Li/Li^+^) using a 0.1 mV/s scan rate. To perform the galvanostatic charge/discharge cycle tests, MTI battery analyzers were utilized at various current densities (20, 100, and 200 mA/g) within 1.0–3.0 V (vs Li/Li^+^) on both cast and electrospun electrodes. The electrochemical impedance test was performed within a frequency window of 100 mHz–1 MHz and an AC amplitude of 5 mV using the PARSTAT MC system.

To estimate the chemical diffusion coefficient, the cast electrodes were utilized. For the Randles–Sevick technique, CV tests with various scan rates (0.1, 0.5, 1, 5, and 10 mV/s) were done on the heat-treated active materials. To characterize the Li^+^ diffusion kinetics of the active materials, the galvanostatic intermittent titration technique (GITT) was used. The active materials cells were cycled against the Li metal in CR 2032 coin cells. Therefore, a fixed number of Li^+^ were inserted into the host electrode by applying a constant current pulse of short duration (15 min). The tests were done at 20 mA/g between 3.0 and 1.0 V versus Li/Li^+^ on the cells which have active materials of anatase, TNT, HT-5, and HT-10, separately.

## Results and Discussion

### XRD Characterizations of the Samples

To synthesize TNT, hydrothermal treatment was performed on the anatase powder using a 10 M NaOH solution for 48 h. During the hydrothermal process, Ti-O bonds were broken and a Na^+^-accommodated layered TNT was formed.^18^ Thereby acid washing, alkali ions were substituted with hydronium ions through accurate pH adjustments to obtain elongated TNTs.^19,20^ Further heat treatment aims to take out the existing water molecules in the structure and reach expanded pathways for Li^+^ diffusion.^21^ Based on the heat treatment temperature, various structures such as TNT, anatase, TiO_2_-B, or a combination of these can be obtained.^6,21−25^

**Figure 2:**
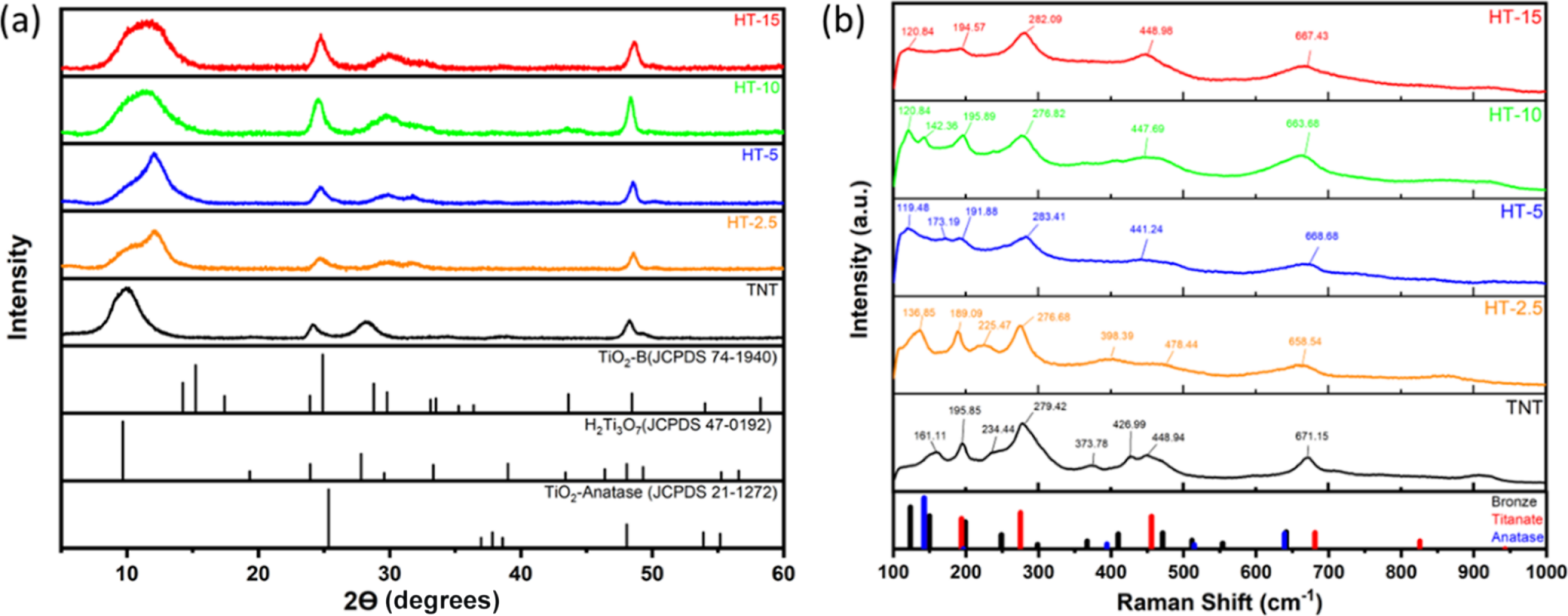
(a) XRD patterns of TNT and heat-treated samples at 500 °C at various durations. (b) Raman spectra of samples before and after heat treatment.

Figure S1 shows the XRD pattern of the anatase precursor. The main peaks are 25.5, 37.8, and 48.0° which correspond to (101), (004), and (200), respectively. After hydrothermal and acid washing, the anatase phase converted into the layered hydrogen titanate structure (JCPDS 47-0192).^26^Figure 2 shows that the structure of TNT and peaks located at 10.1, 24.2, 28.2, and 48.1° are attributed to (200), (110), (003), and (204), respectively.^27^

In this study, post-heat treatment was carried out under an Ar atmosphere at 500 °C. Aiming to reach TiO_2_-B, 500 °C was chosen as the optimum heat treatment temperature since higher and lower temperatures bring about the anatase and hydrogen titanate phases, respectively. Figure 2 also shows the XRD patterns of the heat-treated samples in various durations. HT stands for the heat-treated samples and the number after HT shows the duration TNT was kept at 500 °C (for instance HT-2.5 means standing at 500 °C for 2.5 h).

The in situ experiment done by Zhu et al. showed that heat treatment under air at 450 °C resulted in the disappearance of (200) peak and the generation of new peaks at 14, 15, and 28° attributed to the presence of TiO_2_-B.^22^ In the current study, which was done under an Ar atmosphere, the TNT’s (200) peak exists for all durations. However, based on the various durations, the intensity of the peaks varies. By applying the heat treatment for up to 5 h, the conversion of peaks at 10.1° to the ones at 14 and 15° proceed. For HT-10 and HT-15, however, it was observed that the relative intensities of the titanate and bronze phases (at 2θ ≤ 15°) become equal. The peak at 28° shows a mild bulge for HT-2.5 and becomes intensified by further heat treatment. This peak at 28° experiences a maximum for HT-10 followed by a slight decrease for HT-15. Other peaks existing in the patterns could be attributed to both phases at the same time. This suggests that all the samples are composites of TNT, anatase, and TiO_2_-B. In the literature, this situation is often overlooked since they have common or very close peaks. Moreover, interestingly, XRD patterns of the samples treated under air mainly contain anatase with some portion of TiO_2_-B.^21−24^ Accordingly, Rietveld refinement analyses were performed on the patterns.

Figure S2 shows the refined results for the heat-treated samples. Based on the contribution of each phase (Figure S3), it can be understood that the highest amount of the bronze phase (∼55 wt %) exists in HT-10. Conversely, the titanate amount decreases with the increase in thermal treatment duration. All of the samples showed almost the same amount of anatase phase through the heat treatment.

### Raman Spectroscopy

All synthesized materials were characterized with Raman spectroscopy, as shown in Figure 2b. Based on the references, similar to the XRD peaks, all samples’ spectra obtain the representative peaks of titanate,^28^ bronze,^29^ and anatase^30^ phases. For TNT and HT-2.5, the most intense peaks are around 270 cm^–1^ showing the highest contribution of the titanate phase. However, for HT-5 and HT-10, the most intense peaks match well with peaks related to the bronze phase (∼120 cm^–1^). It is worth noting that there is also a high contribution of the titanate phase in HT-5 and HT-10. HT-15, however, shows that the peak related to the titanate phase obtain the highest intensity. Due to the similarity and overlapping of titanate and bronze phases, it is difficult to decide on the presence of each phase within a specific sample.

### Morphological Characterization

**Figure 3:**
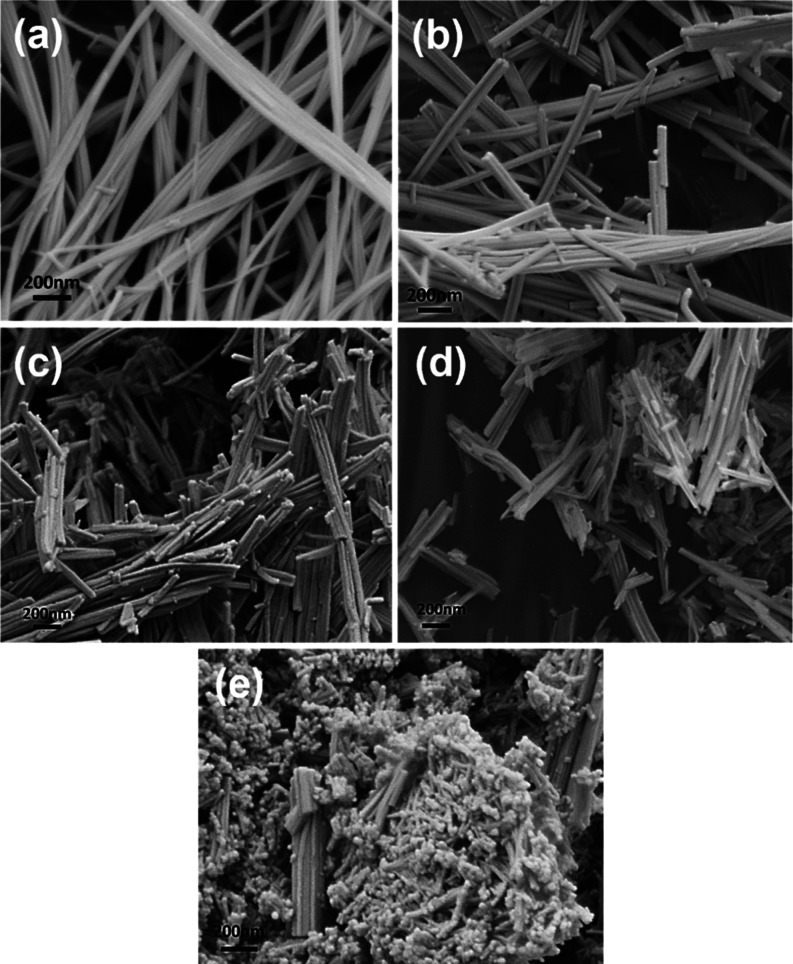
SEM images of TNT (a), HT-2.5 (b), HT-5 (c), HT-10 (d), and HT-15 (e).

The phase and size of the precursor play a significant role to reach a pure TNT since very small particles have a tendency to be agglomerated which leads to a decline in recrystallization rate.^31,32^Figure S4 shows the scanning electron microscopy (SEM) image of the as-received anatase powder. The average size of anatase utilized in this study is about 200 nm. As shown in Figure 3a, after hydrothermal treatment and acid washing, nanotubes with a length of several microns were achieved. For the thermal treatment step, high temperatures cause the collapse of the nanotubular structure which could result in nanosphere morphologies.^21^ As shown in Figure 3b,c, some nanotubes in HT-2.5 and HT-5 are broken; however, the overall nanotubular morphology is preserved. By increasing the duration of the heat treatment to 10 h, the length and width of the structure were remarkably diminished, and there are almost no elongated nanotubes similar to the as-synthesized ones (Figure 3a). As shown in Figure 3e, by increasing the duration to 15 h, spherical particles started to appear.

**Figure 4:**
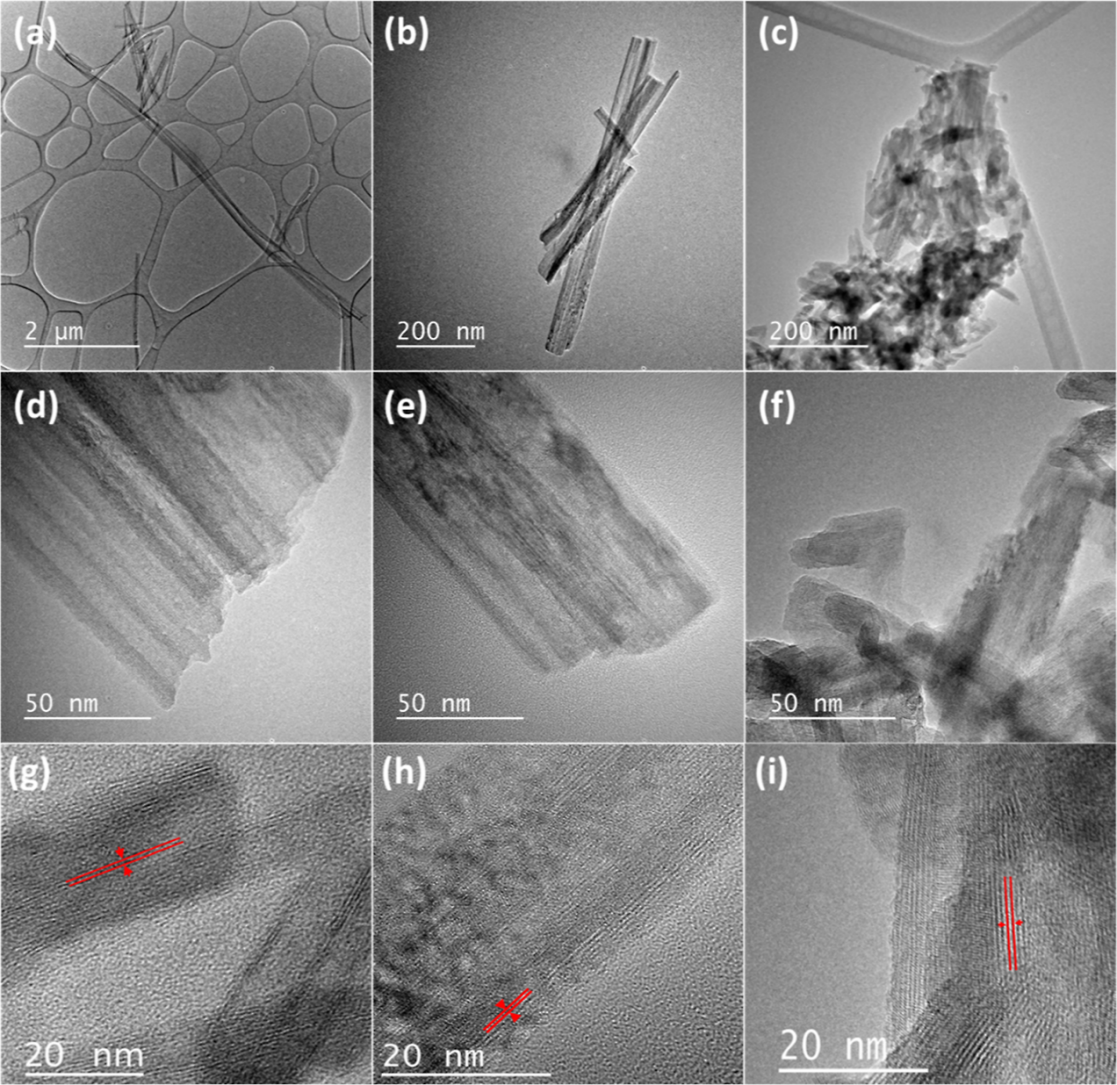
TEM images of TNT (a,d), HT-5 (b,e), and HT-10 (c,f) in two magnifications. HRTEM image of (g) TNT, (h) HT-5, and (i) HT-10.

Transmission electron microscopy (TEM) images of TNT (Figure 4a,d) show a wide range of lengths (2–8 μm) and an average width of about 62 nm. Other studies have also reported that elongated TNTs share similar properties.^19,20^ The TEM images of HT-5 (Figure 4b,e) indicate that the structure maintains its morphology however the average length and width of the nanotubes were reduced to about 380 and 35 nm, respectively. The TEM images of HT-10 show tiny needle-like structures with an average length and width of 20 and 8 nm (Figure 4c,f). Interlayer distances were measured using HRTEM images (Figure 4g–i). Based on the planes parallel to the length of the nanotubes [(200) plane], interlayer distances are 0.81, 0.74, and 0.7 nm for TNT, HT-5, and HT-10, respectively. The obtained distance for TNT was comparable with the other research (∼0.8 nm).^20,33,34^ By increasing the thermal treatment duration, more surface is exposed and this can facilitate the electrode/electrolyte contact. Moreover, the Li^+^ insertion and deinsertion become easier which results in a better cycling and rate capability performance.^21,24^ With the increase in thermal treatment duration, the interlayer distances reach to the (001) plane of TiO_2_-B, which proves the successful conversion to the material.^24^ Thanks to the wider interlayer distances obtained in the heat-treated samples compared to the anatase crystals (almost two times), better Li-ion diffusion and more storage capability can be achieved.^24,35^

### Pore Structure Characterization

**Figure 5:**
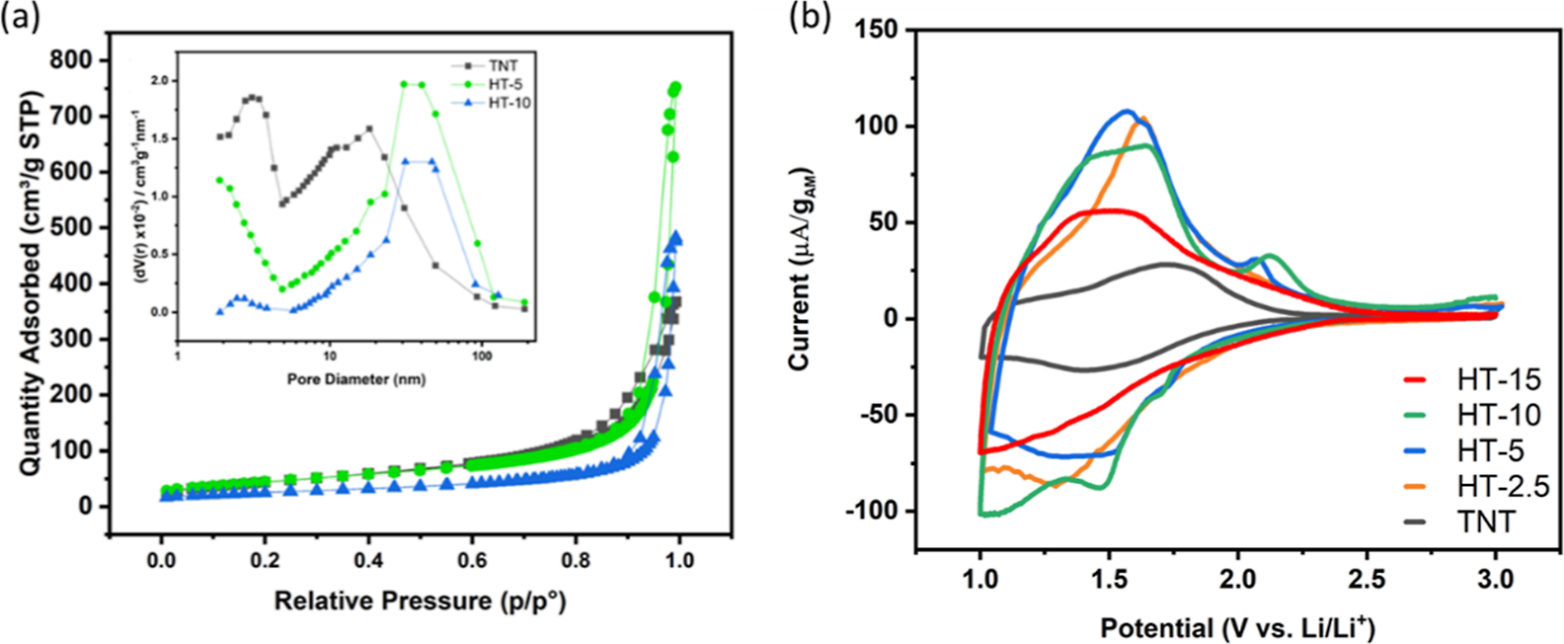
(a) BET isotherm and BJH PSD curve, and (b) CV of TNT, HT-2.5, HT-5, and HT-10.

To better understand the pore structure, BET characterizations were performed. The textural properties of the active material play a significant role in the ultimate performance of the battery. Materials with high porosities and larger specific surface areas enhance the diffusion kinetics by decreasing the diffusion pathways.^20^ As shown in Figure 5, the BET analyses revealed that the BET surface areas for TNT, HT-5, and HT-10 are 162, 161, and 90 m^2^/g, respectively. Therefore, after 5 h of heat treatment, the specific surface decreases remarkably which could be attributed to the shrinking of the tubular structure (Figure 4). However, it should be reminded that after the thermal treatment, the nanotubes become lighter which is ideal for Li^+^ storage. Obtained cumulative pore volumes are 0.57, 1.16, and 0.74 cm^3^/g for TNT, HT-5, and HT-10, respectively. All samples showed type II adsorption isotherms with H3-type hysteresis loops. H3 hysteresis loop found in solids consisting of slit-shaped pores consistent with the TNT’s rolled structure.^36^ The BJH PSD curves show that TNT comprised mesopores with a sharp peak at around 3.0 nm corresponding to the inner volume of nanotubes. Similar values have also been reported in the literature.^19,37^ The mesopore intensity is larger in HT-5 compared to HT-10. Like the shrinking of the interlayer distances, the thermal treatment also reduced the inner pore volumes. On the other hand, interparticle distances increase and pores around 40 nm start to appear.

### Electrochemical Characterization

#### Cyclic Voltammetry

Figure 5b shows the CV of the samples before and after heat treatment between 1.0 and 3.0 V versus Li/Li^+^ at 0.1 mV/s scan rate. TNT cast sample shows a typical CV curve of lithium titanate with anodic (and cathodic) peaks at 1.7 (and 1.4) V versus Li/Li^+^.^19,20^ However, for the heat-treated samples, there are a pair of peaks (the initial and 10th cycle CV of each sample are separately shown in Figure S5). Peaks at 1.7 (and 1.4), 1.6 (and 1.5), and 1.6 (and 1.5) V versus Li/Li^+^ show the deinsertion (insertion) potentials of TiO_2_-B for HT-2.5, HT-5, and HT-10, respectively. On the other hand, the less prominent peaks at 2.0 (and 1.7), 2.1 (and 1.7), and 2.1 (and 1.7) V versus Li/Li^+^ show the anatase delithiation (and lithiation) processes for HT-2.5, HT-5, and HT-10, respectively.^38^ By increasing the heat treatment duration, until HT-15, the distinction and the intensities of the peak pairs become clearer; furthermore, the contribution of TiO_2_-B in the current generation increases, while the anatase-related peaks diminish. Therefore, it can be concluded that at higher durations, during the restructuring of the tubular material, low-range ordering of titania occurs, and its intercalation/deintercalation potential shifts to lower potentials.^35^

#### Galvanostatic Charge/Discharge

The galvanostatic discharge/charge profiles of the samples were obtained over the potential range of 1.0–3.0 V at the current density of 100 mA/g (see Figure S6). The profiles bear resemblance to the Li^+^ insertion/extraction in the bronze phase (pseudocapacitive behavior)^39^ for both the 1st and 100th cycles. It can be observed that by increasing the heat treatment duration to 10 h, except for the TNT sample, the initial cycle’s discharge capacity is improved, 230, 165, 200, 226, and 154 for TNT, HT-2.5, −5, −10, and −15, respectively. Significant capacity reduction in HT-2.5 compared to TNT could be attributed to the interlayer distance reduction due to thermal treatment (Figure 4). The rapid reduction in the capacity of HT-15 could be attributed to its morphological changes (Figure 3) and reduced diffusion coefficient, which will be elaborated on in the following. The first cycle’s irreversible capacity loss is considered one of the disadvantages of the presence of the bronze phase due to the parasitic reactions of Li^+^ with Ti-O and Ti-OH existing in the structure.^39^ As shown in Figure S7, the Coulombic efficiencies in the first cycle are 55, 67, 81, 74, and 82% for TNT, HT-2.5, −5, −10, and −15; respectively. It shows that by increasing the heat treatment duration, the Coulombic efficiency is enhanced. After around 20 cycles, the Coulombic efficiency’s magnitudes become stabilized, and as shown in sub-Figure S7, the longer the duration, the larger the Coulombic efficiency.

**Figure 6:**
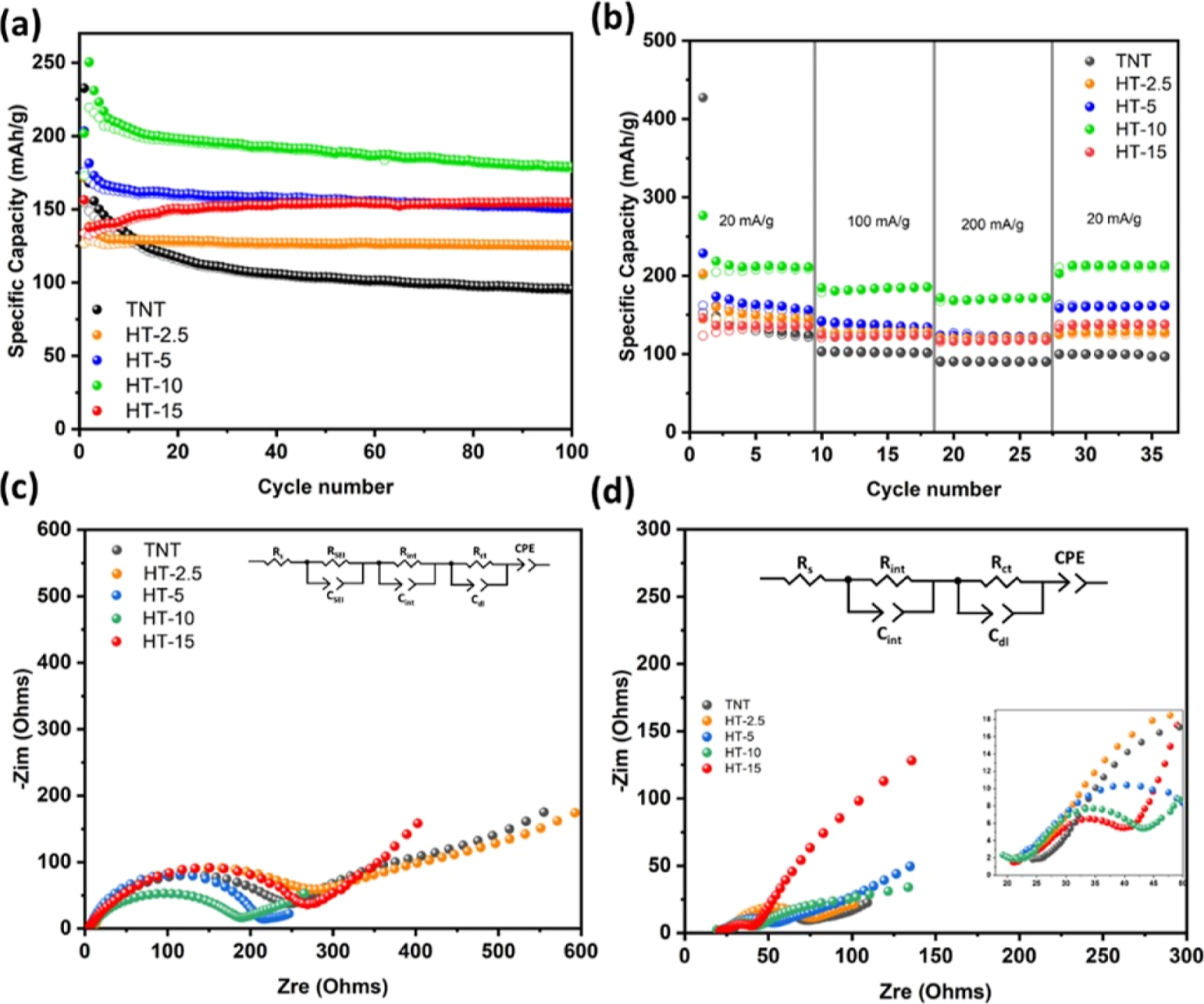
(a) Cyclic and (b) rate performance of the samples with various heat treatment conditions. (c,d) Nyquist plot of the electrodes at their OCP before cycling and completely lithiated after 100 cycles, respectively.

Figure 6a shows the cycling performance of the samples for 100 cycles at the current density of 100 mA/g. The highest obtained capacity is 233, 171, 203, 250, and 156 mA h/g for TNT, HT-2.5, −5, −10, and −15; respectively, which reduces to 95, 124, 151, 179, and 154 mA h/g after 100 cycles. HT-10 obtains the highest capacity which could be attributed to the higher contribution of the bronze phase in the structure (Figure S3) and improved Li^+^ diffusion. The capacity fade rate is 1.38, 0.47, 0.52, 0.71, and 0.02 mA h/g per cycle for TNT, HT-2.5, −5, −10, and −15; respectively. This trend shows a significant improvement in capacity retention after heat treatment. The highest retention belongs to the HT-15 samples which contain higher Coulombic efficiency compared to the other samples.

Considering the rate performance of the cast samples, HT-10 shows superior capacity compared with the other samples. For instance, in the 200 mA/g, the discharge capacities are 97, 131, 145, 180, and 114, for TNT, HT-2.5, -5, -10, and -15; respectively. For HT-10, by a 10-times increase in the current density (from 20 to 200 mA/g), the capacity only reduces around 20% (from 203 to 157 mA h/g).

#### Electrochemical Impedance Spectroscopy

To investigate the electrochemical kinetics at the various durations of the heat treatment, the impedance spectrum of the samples was obtained. Figure 6c,d shows the Nyquist plot of the samples at their open circuit potential (OCP) before cycling and after complete lithiation in the 100th cycle, respectively. After assembly, there is almost no SEI for pure bronze phases; however, due to the presence of other phases, one may consider the SEI layer.^40^ Therefore, for this system, the polarization resistance (*R*_s_) can be modeled in series by a parallel RC combination. An additional RC component was added due to interfacial resistance (*R*_int_) between the electrode/electrolyte.^41^ A double layer capacitance (*C*_dl_), due to the short distance and large surface in porous electrodes,^42^ also exists in parallel with the charge transfer resistance (*R*_ct_) accounting for faradaic resistance. Thereby fitting equivalent circuits to the spectra, the kinetics of the Li^+^ in intercalation and deintercalation could be studied. Details of the fitted values are shown in Tables S1 and S2 for the cells after assembly and after 100 cycles.

According to Table S1, *R*_s_ at OCP is almost the same for all samples; however, after 5 h of thermal treatment *R*_SEI_ reduced remarkably, which could be attributed to the presence of a considerable amount of TiO_2_(B) within the structure (Figure S3).^40^*R*_ct_ is almost doubled after heat treatment due to the reduced interlayer gap of the structure (Figure 4); however, by continuing the treatment thanks to the creation of a higher amount of the bronze phase, the facile Li^+^ transfer can take place. By reaching the HT-15, due to the losing tubular morphologies (Figure 3), there is a significant increase in the *R*_ct_. *R*_int_ shows the trapping of the electrolyte within the porous media and binder.^43^ By increasing the temperature, due to the reduction of the porosities within the system, a lower *R*_int_ is expected.

For the cycled samples, one may estimate a very low amount of SEI formation; therefore, RC related to SEI was chopped from the equivalent circuit. Due to the irreversible hydrolyzation of the electrolyte because of the plethora of −OH and −O surface groups within the titanate layers,^19^*R*_s_ was increased for all samples (Table S2). Generally, *R*_ct_ reduces remarkably thanks to the increased conductivity of SEI layered formed in the initial cycles.^44^ Obtaining more bronze structure could explain the additional reduction of the *R*_ct_ after 5 h of treatment. A similar trend of *R*_int_ for samples before cycling (Table S1) can be observed for cycled samples.

#### Diffusion Coefficient

The chemical diffusion coefficient represents the transport kinetics of mobile species whenever a concentration gradient is applied. Since the diffusion of Li-ions into the lattice of the host electrode is a bottleneck during cycling, the Li-ion chemical diffusion coefficient, , plays a significant role in understanding the kinetic properties of the electrodes.^45^

One way to estimate  is using the Randles–Sevcik equation which relates the swiping rate (ν) to the peak current (*i*_p_)^45^
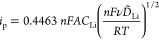
1in which, *n* is the number of electrons exchanged, *F* is Faraday’s constant, *A* is the area of the electrode in contact with the electrolyte, *R* is the gas constant, *T* is the temperature, and *C*_Li_ is the concentration of the analyte. Therefore, to understand the diffusivity of Li in various materials, the electrodes were tested under various scan rates (0.1, 0.5, 1, 2, and 5 mV/s). The resulting CVs (Figure S8) show that the peak current density obtains an increasing trend as a function of the heat treatment duration; however, it experiences a maximum at HT-10 and reduces for HT-15.

To calculate the diffusivity, the anodic peaks were considered. Therefore, *n*, the number of Li-ions taken out from the structures, needs to be estimated based on the amount of the phases (anatase, titanate, and bronze) existing in the samples. In this regard, the lithiation reactions are considered as below^46−48^
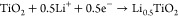
2
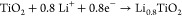
3
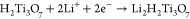
4

Equations 2–4 show the lithiation reactions for the anatase, bronze, and titanate, respectively. Hence, the number of Li-ions that gets into the structure will be 0.5, 0.8, and 2 for the anatase, bronze, and titanate phases. Using the contributing amount of the phases in Rietveld refinement (Figure S3), n was estimated. Accordingly, the diffusion coefficient can be calculated by obtaining the slope of peak current versus scan rate (Figure S9). Table 1 shows the estimated values for the diffusivity of Li-ions within the anode in different heat treatment durations. Similar to the peak current density behavior, HT-10 obtains the highest values of diffusivity which can be attributed to the presence of a higher amount of bronze phase within the structure.

**Table 1:** Calculated Diffusivity for the Samples at Various Heat Treatment Durations (All Calculated Diffusion Coefficients are in cm^2^/S) Using Various Techniques

sample	diffusivity (from CV)	diffusivity (from GITT)
anatase	9.32 × 10^–8^	5.52 × 10^–8^
TNT		2.91 × 10^–7^
HT-2.5	8.52 × 10^–8^	
HT-5	1.67 × 10^–7^	3.72 × 10^–7^
HT-10	3.14 × 10^–7^	3.82 × 10^–7^
HT-15	1.05 × 10^–7^	

Another way to estimate  is GITT. Although in GITT materials are considered as non-porous,^49^ comparison of the results between diffusion coefficients obtained from this method and Randles–Sevik could reveal the compatibility of this method. By assuming that the Li-ions transport is a function of Fick’s second law,  can be obtained using the following equation
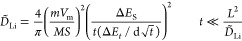
5in which, *m* and *M* are the mass and molar mass of the active materials, respectively, *V*_m_, the molar volume of the anode material, can be obtained by *N*_A_ × *V*_unit_/3, where *N*_A_ is Avogadro’s constant. *V*_unit_ is the volume of the unit cell. *S* is the total contact area between the electrode and electrolyte, and *L* is the thickness of the electrode. Figure S10 shows the GITT results and calculated diffusion coefficient electrodes obtained from anatase, TNT, HT-5, and HT-10. Although this method is Table 1 summarized the apparent diffusion coefficient obtained from GITT. The coefficients with similar order of magnitude have been reported in the literature.^50,51^

Among the all cases, the HT-10 sample shows a higher amount of diffusivity, which matches well with electrochemical kinetics (measured by EIS) and cycling behavior.

#### Electrospun Electrode

Electrospun electrode provides a fibrous structure in which the active materials are embedded within a polymeric interface. Thanks to the presence of this interface, active materials can be accommodated within and out of the polymeric interface in contact with the conductive agent and electrolyte. The superiority of this polymeric interface over the normal cast electrodes other than being freestanding is providing enhanced infiltration of the active materials with electrolyte and better electrochemical kinetics.

**Figure 7:**
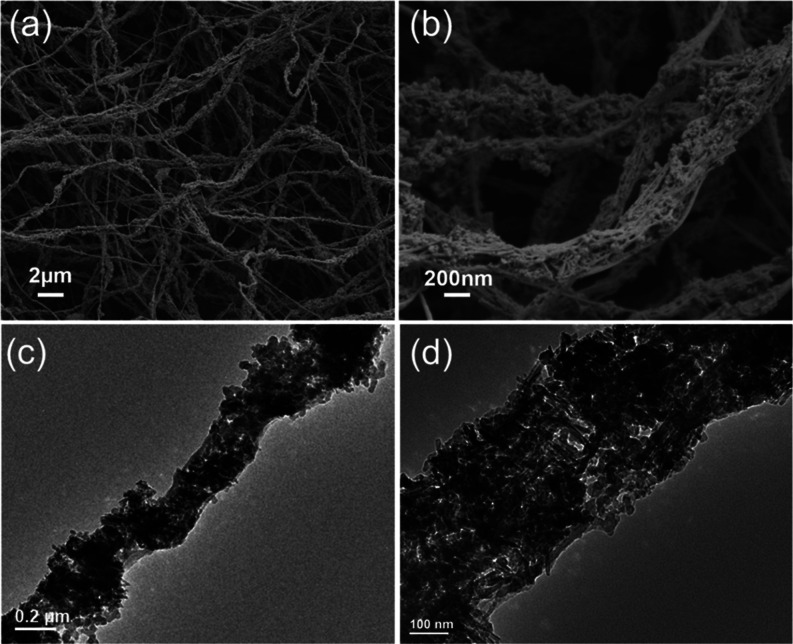
(a) TEM image of electrospun electrode out of HT-10 sample (with two different magnifications), and (b) electrospun electrode made from the HT-10 sample in two magnifications.

In order to obtain the nanofiber-based electrodes, the electrospinning technique is utilized. The morphology of fabricated fibers was investigated using SEM and TEM. SEM images (Figure 7a) show that the majority of the fibers are covered with active materials and CB. They illustrate that the fibers were fully covered with HT-10 nanotubes. Based on Figure S11, the average fiber diameter is around 450 nm. Figure 7c,d shows the TEM image of the HT-10 electrospun electrode. The magnified image illustrates that the fiber is fully covered both with the tubular HT-10 and spherical CB without any noticeable agglomerations. Large diameters of the fibers allow both the longitudinal and transverse accommodation of nanotubes.

To obtain the best compaction while preserving the porous structure, the electrospun electrodes were pressed at various temperatures. Figure S12 shows the SEM images of the samples after the pressing. It can be concluded that pressing at 80 °C gives the best compaction and porosity. The samples pressed at higher and lower temperatures suffer from blocked porosities and insufficient compaction, respectively. Therefore, the electrochemical tests were done on the samples pressed at 80 °C (hot-pressed samples) and 25 °C (cold-pressed samples).

**Figure 8:**
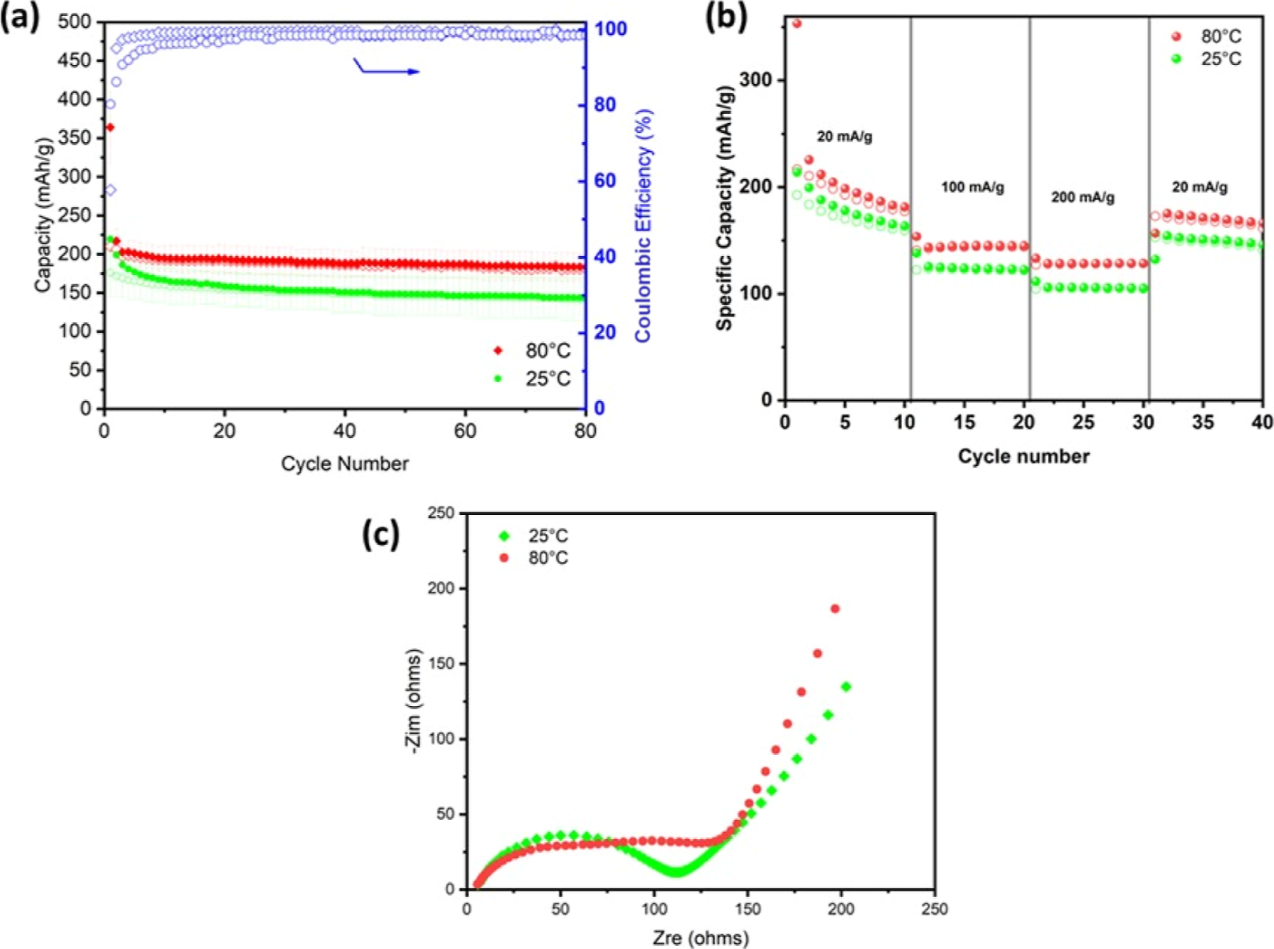
(a) Cycling Performance, (b) rate performance test, and (c) EIS spectra of the electrospun electrodes.

Figure 8a shows the cycling performance of the electrospun electrodes. The hot-pressed sample shows a higher capacity which may be attributed to the better compactness of the material and higher contribution of the active material because of an enhanced fiber volume fraction.^14^ The obtained capacity from the electrospun electrodes is comparable with the ones obtained using the casting method after 80 cycles (Figure 6). Figure 8b shows the rate performance test for the electrospun electrodes. The electrodes obtained stable cycling performance even after a rapid change in the current density. The hot-pressed sample shows a better performance compared to the cold-pressed one, which is more compact and has a better fiber volume fraction. Figure 8c demonstrates that charge transfer resistivity was increased after hot pressing since there was an additional (interfacial) resistance implied to the system compared to the cold-pressed one. However, this compact structure compensates for the little impedance increase in the system.

Table 2 shows the cycling performance of the electrospun electrodes in recent studies. As shown in Table 2, the capacity achieved from the electrospun matrix in this study is higher compared to similar studies. It is worth mentioning that the obtained electrode is free-standing in our study; however, all other studies have fabricated cast electrodes using fabrications followed by a calcination process. To understand the effect of the self-standing electrode without copper current collectors, energy density calculations were done based on 10 mg/cm^2^ active material loading densities^58^ for a hypothetical pouch cell (Table S3). The details of the calculations are covered in the Supporting Information part. In comparison with the cast electrodes, the electrospun electrode showed 22% less mass and this increased the energy density of the pouch cell by 14%.

**Table 2:** Comparison of Electrospun Electrodes

sample	current density (mA/g)	capacity (mA h/g)/cycle #	refs
LTO/graphene	110	180/10	(52)
LTO	88	152/100	(53)
LTO	175	150/100	(54)
LTO	88	140/30	(55)
LTO	175	150/100	(56)
LTO	175	160/50	(57)
this work^a^	100	175/80	

a This electrode is free standing in contrary to other research in the table.

## Conclusions

In this study, for the first time, a controlled thermal treatment process was done on the samples to obtain the highest diffusivity of Li-ion to the structure. After thorough compositional, morphological, and electrochemical optimization, the electrodes with 10 h of treatment showed superior performance. In order to obtain free-standing electrodes, after an arduous optimization process of electrospinning parameters, fully covered electrodes were obtained and characterized. The electrospun electrodes performed well compared to the cast electrodes by delivering 175 mA h/g capacity after 80 cycles at 100 mA/g. The results of electrospun electrodes offer a promising structure for future high-energy applications..

## Supporting Information Available

Anatase powder XRD graph; Rietveld refinement; estimation of contributing phases; SEM image of anatase powder; CV graphs; voltage profile; Coulombic efficiency; EIS equivalent circuit fitted values; CV as a function of scan rate; peak current as a function of scan rate; GITT and diffusion related coefficient graph; fiber diameter distribution; SEM image of pressed samples; and energy density calculation (PDF)
