# Supplementary material for: Electrospun Nanotubular Titania and Polymeric Interfaces for High Energy Density Li-Ion Electrodes
Source: Energy Fuels. 2023 Apr 11;37(8):6197–207. doi: 10.1021/acs.energyfuels.3c00192 (PMC10123667; doi:10.1021/acs.energyfuels.3c00192)
Supplement: Supplementary file 1 — ef3c00192_si_001.pdf [file ef3c00192_si_001.pdf]

Electrospun Nanotubular Titania and Polymeric Interfaces for High Energy Density Li-ion Electrodes

Vahid Charkhesht[a], Begüm Yarar Kaplan[b], Selmiye Alkan Gürsel [a,b], Alp Yürüm [a,b]

a. Sabanci University, Faculty of Natural Science and Engineering, İstanbul, Turkey

b. Sabanci University SUNUM Nanotechnology Research Centre, 34956, Istanbul, Turkey

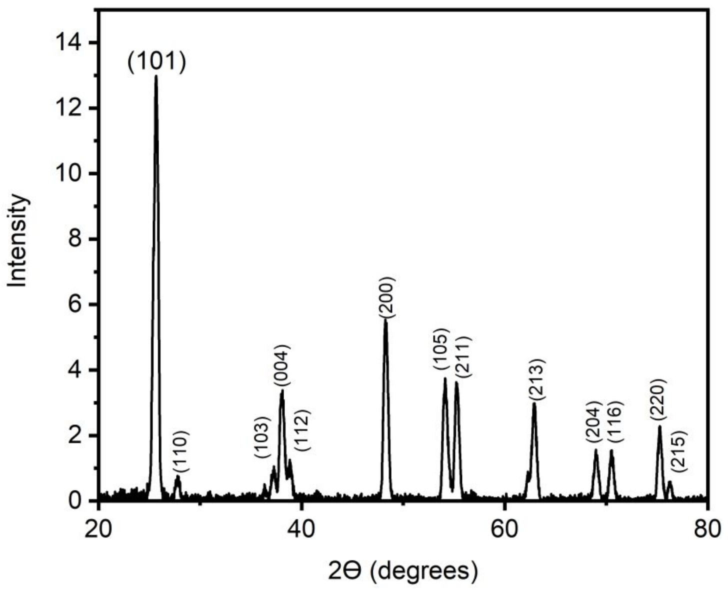

Figure S1. XRD pattern of as-received anatase powder.

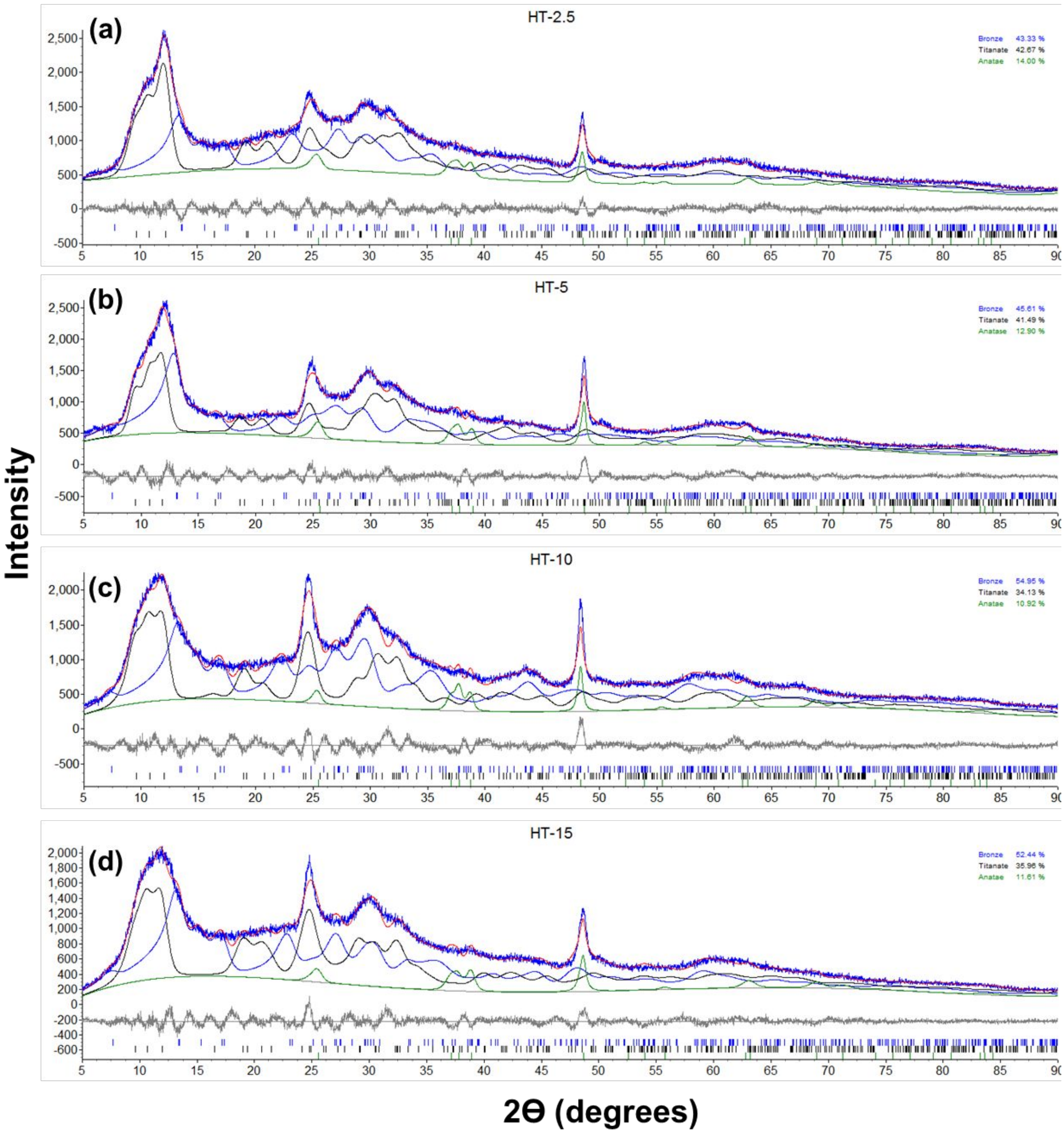

Figure S2. Rietveld refinement of the XRD patterns (a)-(d) corresponding to HT-2.5, HT-5, HT-10 and HT-15 (green, black, and blue show anatase, titanate, and bronze phases, respectively). Red line demonstrates the simulated phase, and grey line shows the differences between simulated and real patterns.

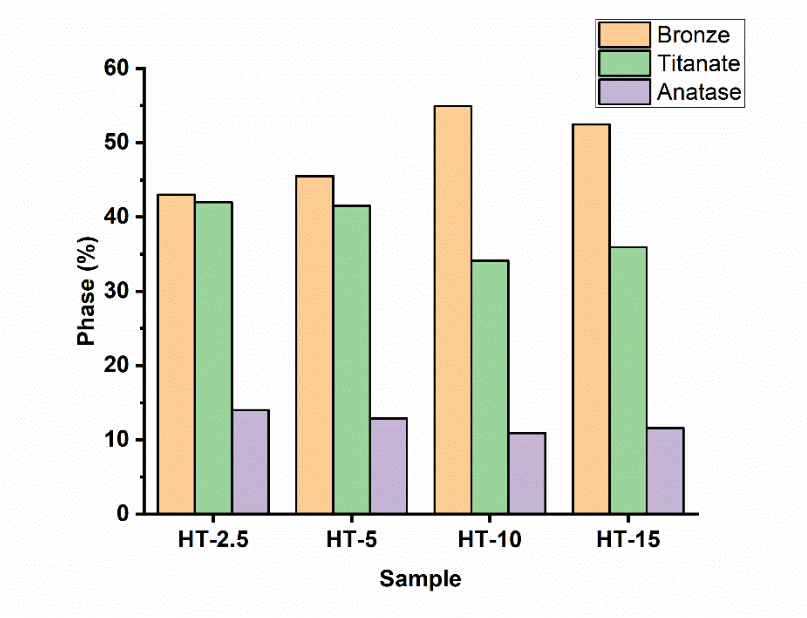

Figure S3. The relative contribution of the phases in heat-treated samples.

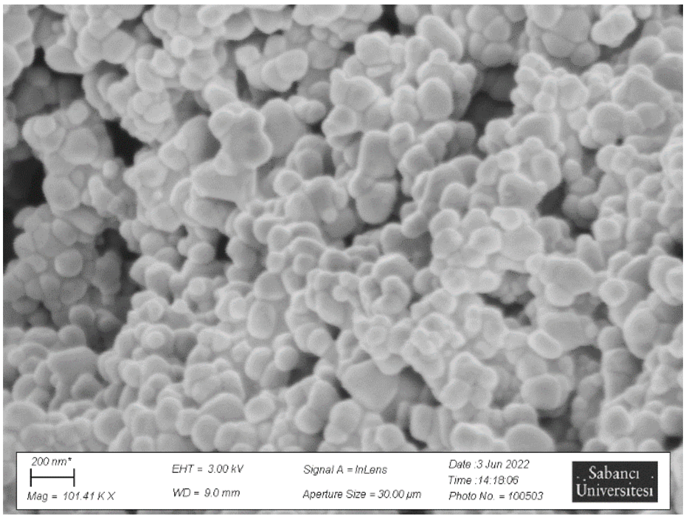

Figure S4. SEM micrograph of as-received powder.

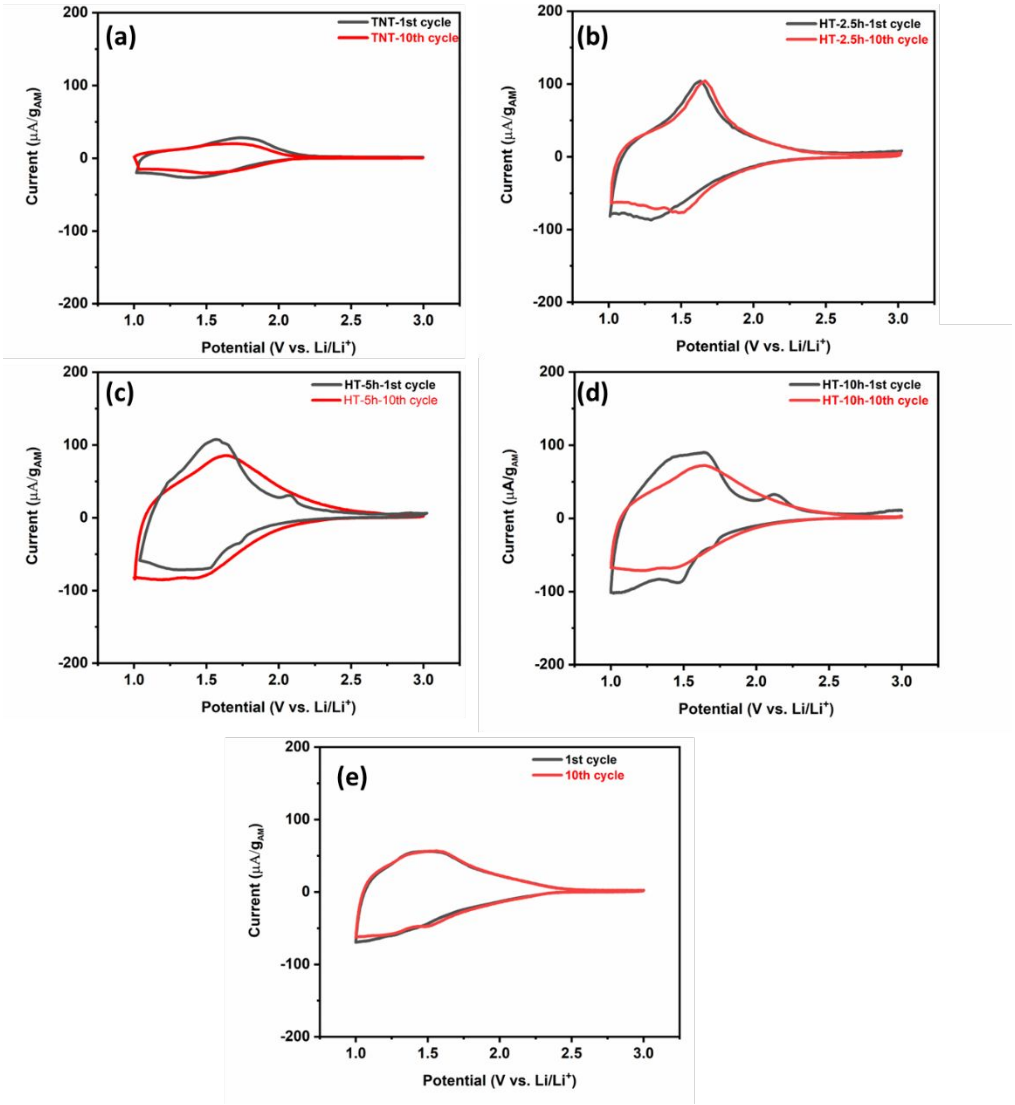

Figure S5. Cyclic voltammograms of (a) TNT, (b) HT-2.5, (c) HT-5, and (d) HT-10 at first and 10th cycles.

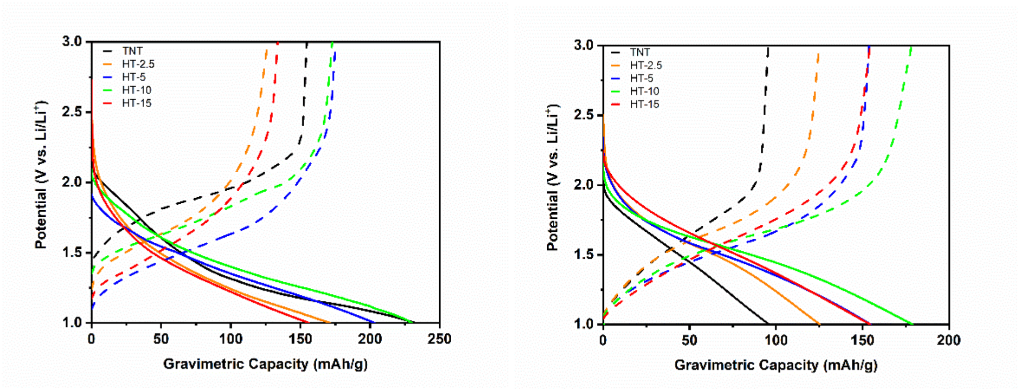

Figure S6. Voltage profile of various samples at different heat treatment durations: (a) 1st cycle, and (b) 100th cycle.

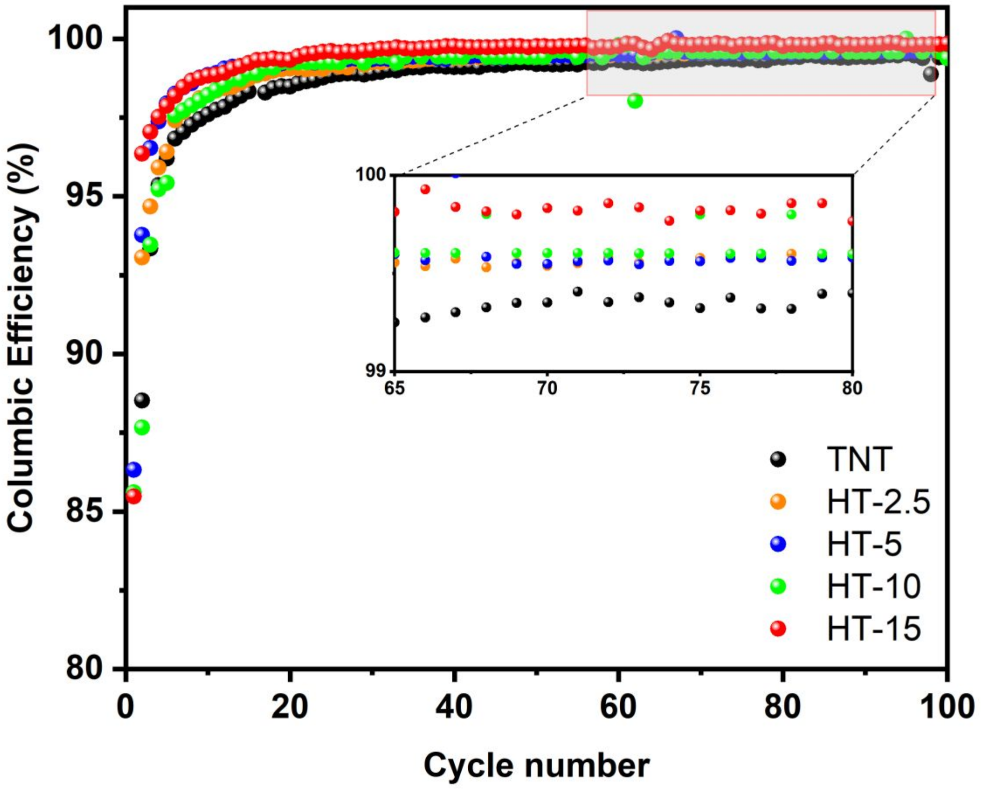

Figure S7. Columbic efficiency of samples with various heat treatment durations.

Table S1. Fitted parameters within the equivalent circuit for the open circuit potential after assembly.

Sample	R_s	R_{ct}	R_{int}	R_{SEI}
TNT	5	123	207	18
HT-2.5	8	250	214	21
HT-5	4	144	45	2
HT-10	7	122	38	4
HT-15	5	201	43	3

Table S2. Fitted parameters within the equivalent circuit for the lithiated state after 100 cycles.

Sample	R_s	R_{ct}	R_{int}
TNT	21	31	15
HT-2.5	22	37	12
HT-5	20	23	4
HT-10	20	12	4
HT-15	19	13	3

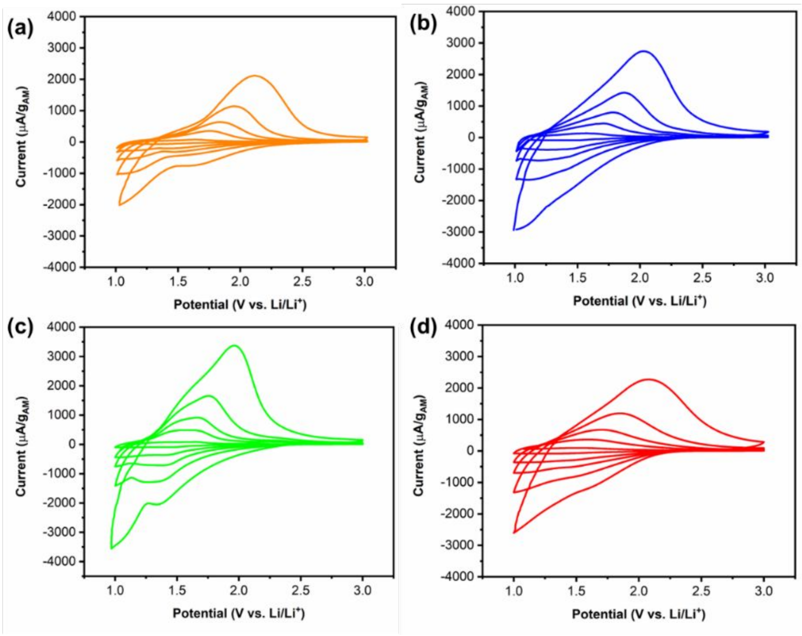

Figure S8. CVs of the (a) to (b) corresponding to HT-2.5 to HT-15, respectively, at various scan rates.

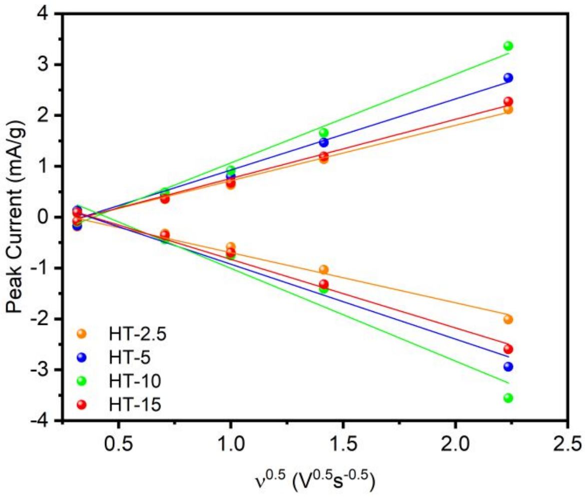

Figure S9. Graph of Peak current vs. square root of scan rate for (a)-(b) corresponding to HT-2.5 to HT-15, respectively.

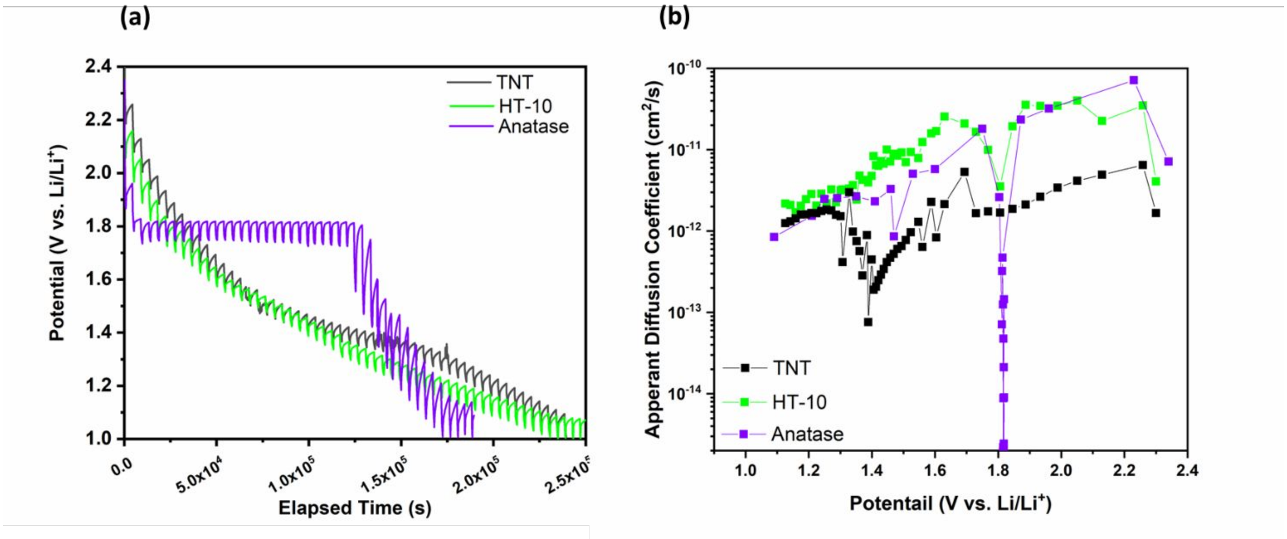

Figure S10. The GITT (Potential vs. time) results for the HT-10, TNT, and anatase electrodes, and (b) related apparent diffusion coefficients.

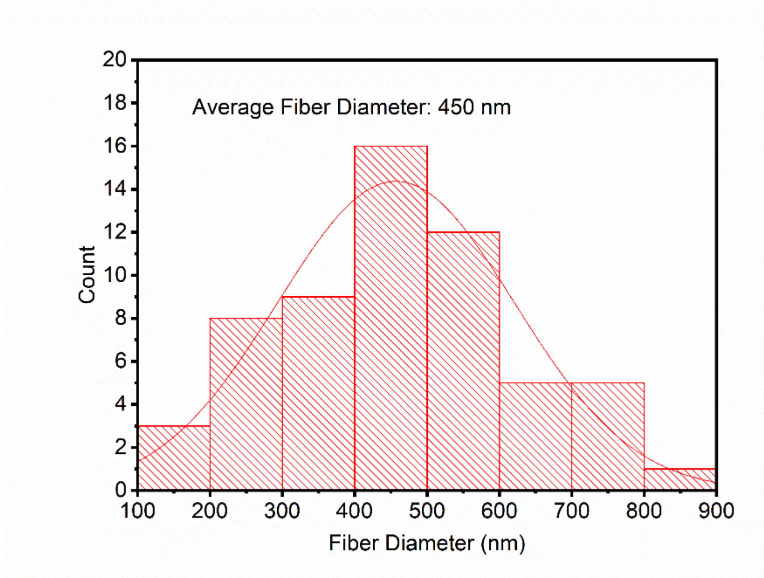

Figure S11. Fiber diameter distribution histogram.

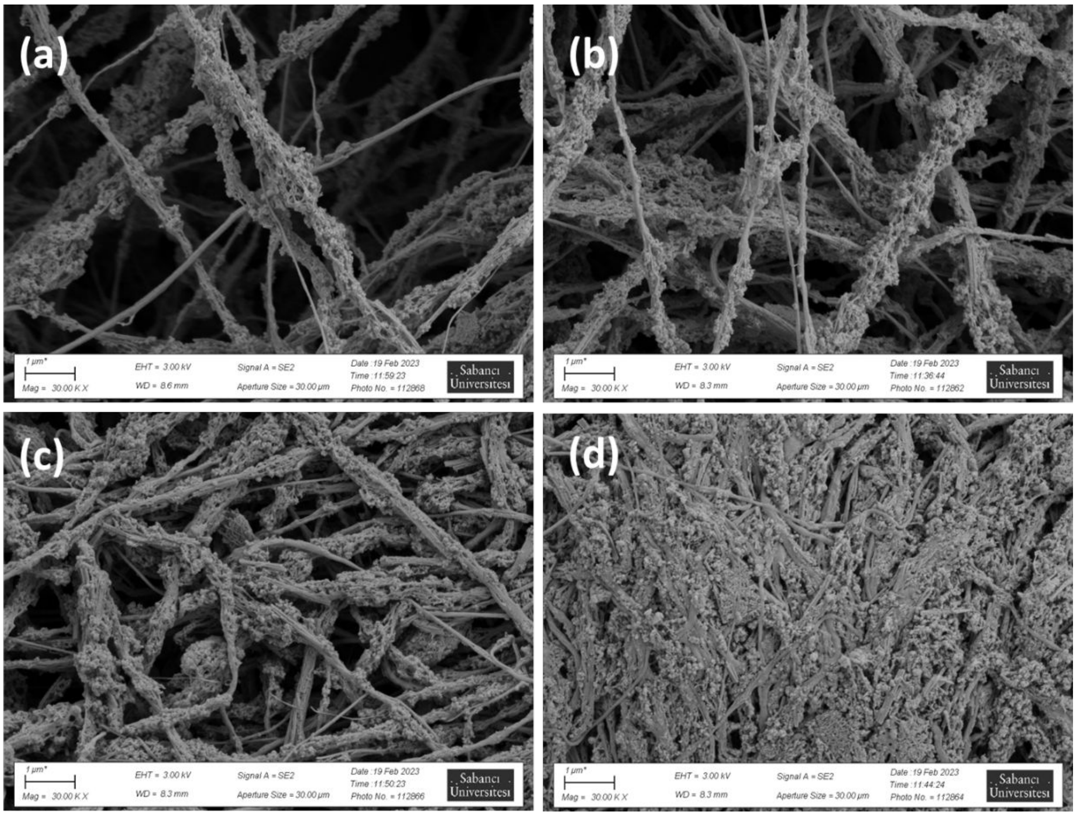

Figure S12. The effect of the pressing condition on the porosity of the fibrous media: (a) No pressing, (b) pressed at 25˚C, (c) pressed at 80˚C, and (d) pressed at 120˚C.

Energy Density Calculation

To compare the efficiency of the electrospun electrods with the cast ones, one may calculate the energy density of the batteries based on Eq. 6:

$$\text{Energy density} = \frac{(V_{\text{Cathode}} - V_{\text{Anode}}) \times \text{Capacity}}{\text{Weight}} \quad (6)$$

in which V is the half-cell potential of the cathode and anode, and weight is the total mass of all parts (including the external case, connector, electrolyte and substrates besides electrodes and separators). In the following, the energy densities of two hypothetical battery comprised of (casted vs. electrospun) anodes versus a commercial cast cathode (LMO) is calculated. Since the folded cells are used in the commercial pouch cells, the repeating units could be considered as the one shown in Figure S13.

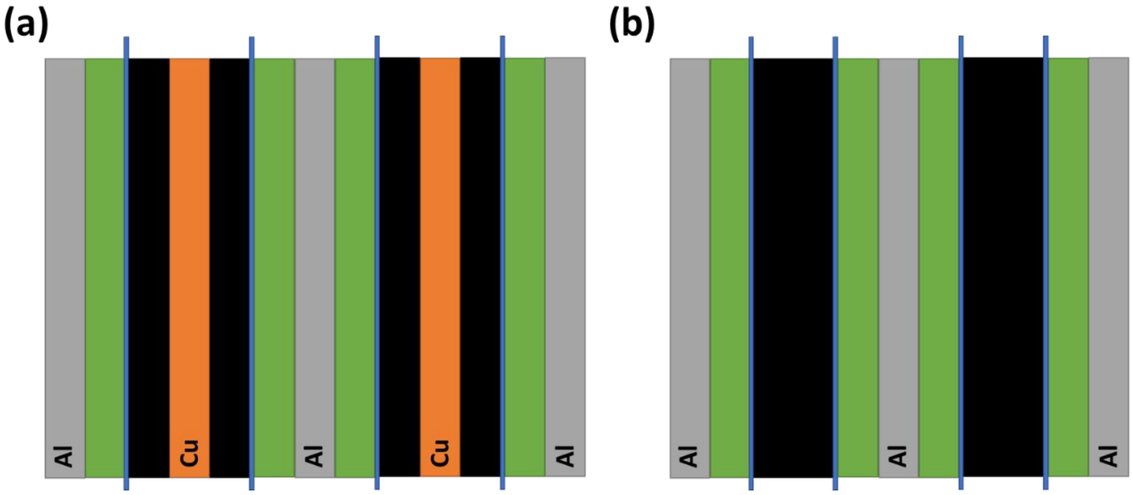

Figure S13. Scheme of the normal pouch cell structure. (a) Conventional electrode, and (b) electrospun electrode (without metallic substrates).

Based on the data obtained from the BatPaC software (Table S3), in a 100Ah pouch cell battery with LTO anode and LMO cathode, there is a 14.4% weight loss with the electrospun electrode, due to removing the Cu current collector. Thus, the overall energy density of the ouch cell increases from 131 Wh/kg to 154 Wh/kg.

Table S3. Energy density calculations regarding the cell types.

Electrode type	$V_{nominal}$ (V)	Capacity (Ah)	Energy (Wh)	Full Weight (g)	Energy Density (Wh/kg)
**Cast**	2.49	100	249	1900.0	131.1
**Espun**	2.49	100	249	1626.4	153.1
